# Hysteria in Connexion with Religious Revivals

**Published:** 1860-01-01

**Authors:** 


### Art. II.—HYSTERIA IN CONNEXION WITH RELIGIOUS REVIVALS.

The diagnosis of hysteria, the precise characters which may be considered pathognomonic of the disease, and the nature of the affections which may simulate it, are all matters that have lately been invested with unusual interest and importance, and that concern a wider circle than the class of purely medical readers. The recent and still progressive awakening of the religious feelings of various communities and sects, the "revival," as it is called, which originated in the north of Ireland, and is now extending itself widely in this country, has been marked, in many instances, by certain manifestations of physical disorder, which have afforded a battle-ground to rival religionists, and an abundance of materials for argument to persons for the most part very imperfectly acquainted with the elements of the subject they undertake to discuss, and the nature of the questions they venture to decide. The physical phenomena witnessed at Belfast and elsewhere have been presumptuously ascribed to the gracious workings of the Holy Spirit; or, on the other hand, to the operation of Satanic agency, by individuals who have no greater right than their neighbours to decide upon the presence of either; or they have been set down as hysteria by those who have never mastered the simplest elements of medical knowledge. Hence an angry conflict between disputants whose words can be brought to the standard of no common measure, and whose anxiety is to support a foregone conclusion rather than to do service in the cause of truth.

Upon the very threshold of an attempt to find essential agreement veiled under these verbal differences, we are met by difficulties which arise from a vague and imperfect nomenclature. In this, as in every other department of inquiry, it is of the first importance rightly to define the terms employed; and we shall therefore endeavour to lay down the sense in which we purpose to use familiar words, and to adhere throughout to one signification for them.

By hysteria, then, we mean to denote a morbid condition produced by some emotion which is denied outlet through its natural channels of activity. Under such circumstances, the force engendered may be supposed to accumulate within the system until sufficiently strong to overcome the active or passive resistance of the patient to its effects; and then to culminate in the production of a paroxysm, or hysteric fit, of a severity and duration proportionate to the original strength of the emotion, or to the exhaustion resulting from efforts to repress it. The fit thus excited is liable to be followed by various kinds of functional exaltation of parts of the nervous system, determined, generally, by the nature of the exciting cause.

Hysteria, in its simple form, if seen from the commencement, is scarcely likely to be confounded with any other disorder. There are cases of true epilepsy, preceded by an aura originating in a diseased organ, which may simulate the hysteria occasionally produced by the contemplation of local morbid changes, or rather by emotions arising out of the existence of such changes; and there are certain stages in many cases of hysteria which may resemble other neuroses; but with these exceptions, little calculated to mislead experienced observers, there are no conditions liable to be mistaken for hysteria.

It becomes a question, therefore, how this word, and its adjective, have been strained to embrace so large a portion of actual pathology? Probably somewhat in the manner following.

A peculiar mental and bodily temperament, being found by experience to involve an increased proclivity to hysteria, has been called hysterical: much as if the constitutional states predisposing to insanity were called maniacal.

An irregular or partial exaltation of nervous function being among the ordinary sequelæ of the hysteric paroxysm—such exaltation, whether physical, as in hyperæsthesia, or mental, as in morbid sensitiveness, has been commonly called hysterical, however induced.

Lastly, inasmuch as malingering is a very frequent concomitant of an advanced stage of one class of hysterical cases, all malingering whatever, in young females, has been confounded with hysteria.

Hence, a large number of infinitely various affections and states, arising from different causes, manifested by different symptoms, and tending towards different terminations, have been combined to form the ordinary conception of a "Protean malady" that, strictly speaking, has no existence. In saying this, we do not for a moment dispute the accuracy, as clinical sketches, of the received descriptions of hysterical knee, hysterical spine, hysteria simulating peritonitis, &c.; but simply wish to affirm that, of two young women who artificially imitate or produce disease, or in whom slight physical changes are attended by prominent nervous symptoms, one may be the subject of hysteria, while the other is not. We protest against the use of any nosological term in a sense so wide as to be utterly indefinite, useless for all purposes of argument or exact description, and valuable only to include a group of phenomena which possess in common one character alone—viz., that they none of them demand active medical treatment. We would, therefore, limit the word hysteria to the paroxysmal affections produced by concealed or suppressed emotion; and the word hysterical, to the various morbid states or actions by which hysteria may be succeeded. It must be admitted that we want a convenient term by which to denote the exalted nervous sensibility that obscures or complicates the diseases of certain individuals, and that involves a marked liability to hysteria; but it is, nevertheless, a perversion of language to call this sensibility, or the diseases it may complicate, hysterical, in a person in whom hysteria has never been developed.

As far as regards the revivals in the north of Ireland, there seems to be an universal consent that any hysteria which may complicate them is in itself a great evil, to be resisted in every possible way. Concerning the *word* we find unanimity among persons of every shade of opinion; and we must approach the thing signified before diversity becomes apparent. That many among the clergy should be slow to recognise hysteria, and, when it is pointed out to them, should cling to a hope that the diagnosis may be erroneous, is a result to be expected from our knowledge of human nature. But that medical writers should gravely argue the question, What is hysteria? with intent to show that it is nothing sufficiently tangible to be discovered and identified with certainty in any case; or that they should refer to the "troubled countenance" of Belshazzar as a phenomenon analogous to the scenes lately enacted in some of the meeting-houses of Belfast, is a matter less easy to explain, and far more painful to contemplate. The revival of religion is no mere local outbreak; and appears likely to display itself as no transient enthusiasm. On various occasions, in former times, such revivals have been kindled; often to serve, early in their progress, as stages for the self-seeker, the vicious, or the profane, and eventually to be trampled under foot by the outraged moral sense of the communities among whom their manifold corruptions have been displayed. In order that the present movement may furnish no parallel, in these respects, to so many that have preceded it, the greatest caution is required from all who can in any way, by writing, speech, or thought, influence its course, or guide the operations of its leaders. If such caution be not exercised, or if, having been exercised, it be relaxed; if the unspeakable blessing of a general turning of the public mind towards God be not used under a deep and watchful sense of responsibility, then old scenes will be acted anew. The sobriety of the Christian pulpit will too surely give way to the rhetoric of the tub; the practicality of Christian doctrine to the imaginative creations of the fanatic; the contrition of the penitent to the performances of the hysterical; and the sincerity of the pastor to the greed of the hypocrite. It can scarcely be doubted that, in former revivals, the first step downwards has been the cultivation of hysteria; and it is the first step that costs. Convulsions and catalepsy, trances and visions, are easily enough produced by some of the weakest, and some of the worst, of womankind; and, when these conditions are recognised as titles to sanctity and signs of grace, when they are seen to be the means of obtaining notoriety and profit, the supply will never fall short of the demand. Magdalens without shame, penitents without humility and without amendment, soon, under such circumstances, afford evidence of the value of the work; and the sober and devout shrink from any confession of their feelings or any record of their experience, lest they should be confounded with the frantic teachers or the questionable devotees of the camp meeting or the hill-side. Such has been the history of many a religious movement, which, at its commencement, promised fairly; but which served only to produce a reactionary coldness, deadness, and formality, wherever its influence was felt. Under the belief that the public mind of this kingdom is at present deeply stirred, and that the results of this impression will depend, mainly, upon the manner in which it is used, we purpose to offer some observations upon the nature and progress of hysteria; in the hope that they may assist to prepare the clergy, and, to some extent, the medical profession also, for dealing at the right time, and in the right manner, with any nervous disorders that may be developed during the course of religious ministrations.

In order to accomplish this object, it is necessary to direct a cursory glance towards those agencies, so pre-eminent among the active powers of man, which we describe collectively as the emotions; and briefly to consider the ends they are intended to fulfil, and their normal and abnormal effects upon the economy. For these purposes it is sufficient to point out that an emotion consists essentially of an idea, either sensational or intellectual, linked with a feeling of pleasure or pain. By a process known to us only through its effects, this combination is able to develope or liberate nervous force sufficient to commence and sustain appropriate action, and bearing a definite relation of quantity to the strength and the duration of the passion that is aroused. Meagre as such an account may seem, when compared with the number and variety of the feelings that chequer human life, it still contains all the essentials of their strength; and it is when the force engendered can neither be self-contained, nor expended through safe and proper channels, that the phenomena called hysterical are displayed.

In order to render this clear, it may be observed that every emotion may be either contemplative or active, according to the degree in which it is developed; and that many of the emotions experienced by every individual never reach the stage of activity at all, but exhaust themselves in the very act of directing the consciousness upon their own existence. The emotion is felt, and the mind is roused out of torpidity or indolence, to dwell upon it for a few moments; after which the impression fades away. In other cases, the feeling, instead of disappearing, gathers strength and intensity with the attention that is paid to it; and (being attended, it may be presumed, with a larger development of force than can be employed in maintaining that attention) calls imperatively for the performance of some action which the circumstances require or suggest. It would be beyond our present scope to inquire into the nature of the peculiarities, either inherent or acquired, that determine the relative force of various impressions in the production of emotion; but the fact is patent to daily observation that, in every person, certain feelings, whenever excited in his mind, exert an immediate control over his actual conduct; while others operate no farther than upon his consciousness. The differences of character, indeed, that are witnessed in the world depend, very greatly, upon differences in the nature of the feelings that are thus habitually active or passive in the case of each individual.

In considering the nature of the activity that the emotions prompt, it is necessary to enlarge our sphere of observation, and to take the lower animals into account. By doing so, the phenomena are presented to us in their simplest forms and combinations; and it becomes manifest that nearly all pleasurable emotions require various kinds of physical activity for their full fruition and enjoyment; while painful feelings require similar activity, in order to remove, or to withdraw from, the causes that excite them. The emotions of sex will serve to illustrate the former kind, and the agonies of fear or hatred the latter; each producing action, either for the possession of the beloved object, or for the mitigation of the pain experienced while the emotion is unrelieved. On the one hand, attainment and possession, on the other, avoidance or removal, may therefore be regarded as the ends towards which emotional force must, in almost every case, be naturally and primarily directed.

In the brute creation, moreover, it is manifest that these ends must frequently be essential, either to the safety of the animal, or to the propagation of the species; for both of which the emotions are plainly intended to provide. The right direction of the force developed is therefore secured "by laws written upon the nervous pulp." The emotions work out their own fulfilment with unerring certainty; and in all cases govern the conduct of the animal. They are expended either upon the motor nerves, through (or by the aid of) the cerebellum, so as to produce co-ordinate muscular movements; or upon the ganglionic nerves, increasing or modifying secretion, or providing a more abundant supply of blood to any parts that may require it. Besides these actions, there are some that, as far as we know, are simply expressive and preliminary, such as many movements of the tail, or of the hairy covering; which, if apparently objectless in themselves, are commonly a prelude to others, and serve a useful purpose by giving warning of the intentions of the animal. Lastly, under strong emotion, when appropriate action is either impossible, or when, having been performed to the limit of the powers of the organism, it is inadequate to afford relief, the lower animals frequently give utterance to a very remarkable cry, unlike the sounds which they naturally emit. The conditions can only be fulfilled by the presence of imminent danger, when escape is rendered impossible, either by fatigue or by physical obstacles. An exhausted hare close before the hounds, a toad or frog pursued by a snake, and many other creatures under similar circumstances, will shriek in a frightful manner; as they will also do, according to M. Brown-Sequard, upon mechanical irritation of that part of the brain called the calamus scriptorius. The cry referred to is probably due to the operation upon the nervous centres of emotional force for which there is no other outlet; and must be carefully distinguished from the many sounds which would be included in the general category of appropriate co-ordinate muscular movements. To the latter must be referred all ordinary cries of pain or distress; the calls between individuals of different sexes, or between the dam and her offspring; and those which most animals originally gregarious will utter upon slight provocation, to serve either as a summons or a warning to their companions.

In the human subject, during infancy and early childhood, the emotions govern the organism to a very great extent. But, as life advances, very remarkable changes in the nature of their influence may be observed.

In the first place, the natural emotional acts that, with reference to brutes, we have called appropriate, would cease to be appropriate in men and women (that is to say, they would defeat themselves) in any condition of life raised above that of the lowest and most brutalized savages. Hence man learns the necessity, and hence the human organism possesses the power, of abstaining from these acts under many circumstances, and by many different methods, of which three are frequently to be observed.

The first of these depends upon the fact that the faculty of judgment and the power of volition afford, in themselves, sufficient employment for many emotions of a highly active character. Every man either possesses, or by timely exercise may obtain, the power to place the sources of all emotion under subjugation to his intelligence and his will; so that the force of his passions is exerted in exciting and sustaining the activity of the hemispherical ganglia, and, partly, perhaps, in giving effect to their commands. In this way, it is not only possible, but common, for the intelligence to supersede the original emotion by one wholly opposed to it; as in cases where the natural disposition to revenge an injury is overruled by the dictates of Christian duty.

Infinitely below the power to control an emotion at its source, but still almost exclusively human, is the power to control it in action for the sake of more certainly obtaining the end to which it points. An emotion that is thus treated is commonly greatly strengthened by the continued direction of the thoughts upon it; but it seldom exerts any hurtful influence upon the body, unless finally and entirely disappointed.

Still lower in the scale stands the direct influence of volition upon the muscular system; an influence that is greatly strengthened, in civilized life, by the handicrafts, or varieties of manual industry, that are almost universally practised. In many persons whose powers of reason and will have not been developed by cultivation, and in whom there is nothing to oppose the growth, or to govern and direct the course of the feelings, there is yet a great capability of suppressing their outward manifestations by the simple expedient of holding the body at rest. When this is done, it becomes a trial of strength between the organism and the emotional force; which, constantly accumulating, and denied outlet through natural channels, is apt to burst forth through others. It is this action which realizes our conception of hysteria; and, in order to understand it, it is necessary to inquire to what effects the term "unnatural" may be applied.

In the human species, to which is given, almost universally, the power to arrest emotions at their outlet; and, as a result of culture, the power to control them at their source, we find that the former seldom becomes complete unless the latter has been attained. In females generally, and in the young of both sexes, the facial muscles, the facial circulation, and the lachrymal glands, are prompt to exhibit feelings which do not otherwise receive relief; and it is difficult to regard expression, pallor, blushes, or tears, in any other light than as safety-valves, expending in harmless action a force that would be hurtful if retained. As the body strengthens, it would appear that the nervous system of organic life is the first to acquire the power of resisting with impunity feelings that are only moderately intense; so that tears or blushing can no longer be excited by trifling causes: and, with complete control over the source of the emotions, there may be gained in time complete control over their manifestations through the countenance. This may be regarded, however, as among the last and most exceptional of volitional attainments; and the actions which we have enumerated are too frequent, as well as too plainly beneficial, to be considered morbid in their general character; although they may sometimes become so by reason of the degree in which they are manifested.

Changes affecting the circulation through the central organ appear to occupy a debateable ground between the normal and abnormal effects of feeling. The force which operates through the ganglionic system is probably intended to stimulate secreting organs; and also, by its action upon the heart and arteries, to supply an increased quantity of blood to any parts, either glandular or muscular, that may be called into activity by the circumstances of the particular case. To a certain degree, therefore, operations of this kind must be considered natural; as when palpitation of the heart, under the influence of fear, is an evident preparation of the system for the active efforts of flight; or when increased salivary secretion is excited by the perception of agreeable food. It is worthy of remark, moreover, that an expenditure of nervous force upon secreting organs commonly brings a hysteric fit to its termination; by providing, we may suppose, natural channels of escape for an agency that was producing morbid effects while such channels were closed. In slight cases of hysteria, weeping will often afford the relief required; and, in more serious cases, a profuse flow of urine commonly follows immediately after the paroxysm. It is obvious, from analogy, that the secretion of urine must be the means of terminating the fit; although the discharge from the body is delayed by the office of the bladder as a containing viscus. From these instances, and from many similar ones, we may conclude that the ganglionic system is one of the natural outlets for the discharge of emotional force; and that the effects of such discharge cannot properly be called morbid, or hysterical, in their nature; although they may oftentimes be injurious when excessive or perverted. For example, strong emotion has been known to produce sudden death by paralysing the heart; to change entirely the character of a secretion formed under its influence; or to destroy the vitality of the secreting organ. It may be conceived, at least as possible, that the difference between such cases and ordinary ones depends mainly upon the amount of force that is suddenly developed; and the parallel case of a discharge of electricity, producing, according to its strength, either moderate stimulation or complete destruction of life, may be cited in support of the opinion.

The nature of emotional muscular actions may be tested, with tolerable certainty, by observation of their tendencies; and, if these are appropriate to the kind of feeling that is excited, the movements must not be considered hysterical. There are many examples of persons who, under the influence of terror, have performed actions in no way guided by volition, but adapted to secure their safety; while, when terror produces hysteria, the attendant movements are objectless or convulsive. We are acquainted with a gentleman who, having only travelled by railway three times in his life, has every time been so unfortunate as to select a train that sustained a terrible accident before he left it. On the last occasion, he extricated himself from the wreck of broken carriages, and, without heed to the necessities of the wounded, took to his heels. He was about three miles from home, and went thither, across country, at racing speed, never stopping, or looking behind him, until he sank exhausted in his own entrance hall. This was not hysteria, but the natural and appropriate action of a frightened brute; and only inappropriate in a man because the human volition ought always to control the human feelings. Perhaps the presence or absence of the co-ordinating influence of the cerebellum might often be taken to determine the nature of the movements in a doubtful case;—that organ being probably the rightful channel through which force is conveyed to the muscles as a result of sensorial changes.

In considering whether any given action be appropriate, it is necessary to look beyond the ordinary range of merely human operations. As in the instance cited above, the predominance of emotion over will lowers man to the level of the brutes; and may occasion things to be done which, seemingly objectless, would yet fulfil important purposes at a certain point in the animal scale. Instinctive actions are not controlled by outward circumstances; and it is a sufficient explanation of defecation or vomiting under the influence of terror, to remark that these acts are the invariable precursors of flight in many beasts of chase; while in some animals, as the polecat and the cuttle-fish, they afford an additional and important provision for escape. The fœtor that is a weapon to so many creatures, and that appears only to be developed when a weapon is required, may be taken to explain how it is that the emotions sometimes operate, as it appears hurtfully, to change the character of various secretions.

The actions already referred to—viz., co-ordinate muscular movements, appropriate to the circumstances, either evidently or by analogy, increased or modified activity of the circulation, and increased or perverted secretion, are, therefore, by our definition, excluded from the phenomena that should be called hysterical; even though they may be clearly the effects of emotion, and may, in some cases, be prejudicial, or even fatal.

When, however, an emotion is very strongly developed, and is denied access to its ordinary channels of discharge, its first action is upon the sensorium, and tends to increase indefinitely that contemplative stage which is a necessary prelude to exertion. In this way it produces a state of morbid self-feeling, self-consciousness, or introspectiveness (as it is indifferently called), which represents the essential moral phenomenon of the hysteric state.

It will readily be apprehended that no distinct line can be drawn beween natural and hysterical self-consciousness; for the reason that the contemplative tendencies vary, within considerable limits, for all classes of feeling. It is equally manifest that, in every case, there must be a certain amount of emotional stimulus that would turn contemplation into action, unless restrained from doing so by the operation of the will.

This amount of stimulus being applied, and the will being exerted merely to hold the body at rest, the state of self-consciousness may next be intensified and prolonged up to the limit of the sensorial capacity for such exertion. In the case of a feeling that is frequently or habitually indulged, while at the same time its proper out-going is restrained, the force excited will often be confined to the sensorium during many successive occasions of indulgence, and will render that organ excessively prone to respond to the call of the particular emotion, or of its immediate kindred. For instance, the intense contemplation, during childhood, of emotions of terror excited by the narratives or threats of a nurse, will infallibly, and very considerably, increase the control of fear over the organism.

In some cases during the first accession of an emotion, in others after many periods of recurrence, the limit of sensorial or contemplative power will be fully reached, while as yet the force developed is not expended. Under such circumstances the force finds its outlet through various channels; and its operations constitute the physical phenomena of hysteria.

It is worthy of remark that these phenomena are often initiated by some trifling circumstance that forcibly arrests the attention, and thus diminishes the sensorial capacity for attending to the feelings. It is very common for an hysteric fit to commence at the moment when a period of reverie is broken by some sudden call upon the outward sense of the individual.

The precise nature of the physical phenomena will vary with the differences of different organisms. We have seen that, in the case of the lower animals, a peculiar and most piteous cry usually attends the development of emotional force in quantity too great for the vital powers; and a cry, similar and probably analogous, is often the immediate precursor of a hysteric paroxysm. Upon this cry usually follow bodily movements, not co-ordinate or appropriate, but objectless and convulsive, affecting chiefly the extremities, the throat or gullet, the eyeballs, or the eyelids. We say chiefly, because such movements are of no absolute necessity, and occur in no determinate order: so that the presence or absence of "globus" (*i.e.*, a choking sensation, as of a ball rising in the throat,—the effect of local spasm), is not conclusive with regard to the existence of hysteria.

In the case of an emotion suddenly aroused, and rapidly culminating, the muscular movements are usually succeeded, before long, by active secretion, either renal or lachrymal; and the paroxysm is brought to a speedy close. The contemplative stage has been short, and the feeling has, so to speak, quickly overflowed the sensorium, without having time to control or modify its habitual operations. In cases of an opposite kind, where the paroxysm follows intense or protracted contemplation, the same degree of relief is not afforded; and the physical phenomena, when they have moderated an excessive or unbearable degree of tension, are commonly followed by some form of somnambulism. In other words, a convulsive fit does not suffice wholly to divert the emotional force from the sensorium; but merely preserves that organ from being altogether paralysed or destroyed by excess of stimulation

It would be tedious to enumerate the possible varieties of the somnambulistic state. There are two of these—namely, trance, and ecstasy, that have most bearing upon our present purpose.

The state of trance can scarcely be distinguished from ordinary sleep, excepting by being more profound. It is characterized by torpor, as far as regards all impressions conveyed through the organs of sense: while, at the same time, the sensorium is cognizant of a train of ideas, or dream, suggested by the active emotion, and coincident with the habitual course of contemplation concerning it.

In ecstasy these conditions are slightly modified, so that the sensorium is percipient of impressions from without, if they harmonize with, or are even associated with, the dominant feeling. There is also some degree of co-ordinate reaction upon the muscular system; so that the course of the dream is indicated, more or less, by appropriate speech and gesture; and the influence exerted upon it by suggestion can be readily observed and demonstrated.

The immediate termination of these states, convulsive or somnambulistic, is necessarily in a period of weakness and exhaustion commensurate with the nervous force put forth; and to be recovered from (if at all) only by the gradual operation of circumstances favourable to strength of body and repose of mind. Their ultimate tendency, is to place both the will, and the physical organism under the government of the emotions; and so to degrade humanity below the level of the brutes that perish.

We say "below the level," advisedly. The passions of the inferior animals are regulated in amount and guided in operation by the hand of unerring Wisdom, so as to produce their due and desired effect in the economy of creation. The passions of mankind, when emancipated from the control of volition and intelligence, are apt to riot in the most unbridled licence; and it is to be observed that the victory of one emotion over the will is the victory of all, of the emotional state generally over the volitional. This is an inversion of the proper order of the human faculties, a subjugation of the spiritual to the animal element in the human organism. As such it may be traced in its effects, producing all shades of perverted belief, from infidelity or credulity to insane delusion; all varieties of perverted conduct, from sensuality or depravity to insane ferocity.

Now if we compare the foregoing account of hysteria with recent actual occurrences, we shall find a complete and remarkable coincidence between them. Starting from the fact that (in a community of persons mostly uneducated, mostly in the condition of imperfect physical tone induced by mill labour, and mostly possessing a dexterity in handicraft that would enable them to exercise a great degree of control over their muscles), an unusual interest has been stirred up with regard to the things which concern eternal life; it may next be observed that this interest has been made a means of attracting large numbers of individuals to crowded places of worship, where they have been excited by the most energetic denunciations of their own lost and fallen state, as well as by threats of the Divine vengeance impending over them. It must be remembered that these denunciations and threats have reference, in the creeds in which the revival took its origin, not to actual, but to original sin; and that hence they were calculated to excite emotions of the utmost terror and despair, from which no way of escape would be pointed out by the preacher along the path of duty. The devotees would be instructed to wait for the Spirit of grace to descend into their hearts; to wait, not in action, not in striving after a holy life, but in the contemplation of their own imputed sinfulness and impending destruction. The feelings hence arising would be intensified by the sympathy of surrounding numbers, would be sustained by the iteration of the preacher, would be directed at no practical aim, would be held off from the muscular system by a sense of the decencies of public worship, as well as by the reflection that bodily flight affords no relief from mental terror. In a congregation thus situated there will soon be an individual whose power of emotional self-consciousness has reached its limit, while the emotion is sustained by the awful words—the repetition of hell!—hell!—hell!--issuing from the pulpit. Then will come the hysterical cry, succeeded by the hysterical convulsion. When the convulsion abates, if the sensorium have been saturated by terror, by an anticipatory self-feeling of the torments of the damned, this feeling will display itself in action, prior to the return of consciousness as regards ordinary outward impressions. Broken words and imperfect actions will indicate the ruling fear; will have reference to Satan, and to flight from or avoidance of his snares. The conversation of bystanders, when relevant to this ruling fear, will serve to guide or modify the acted dream; and their uttered anticipations of coming relief and peace will, as the fear is exhausted, gradually realize themselves. Smiles irradiate the countenance, the Most Holy Name is heard upon the lips, and the somnambulist either awakes in a state of rapture and excitement, or sinks into the sleep that is demanded by her fatigued and enfeebled frame.

In accordance with the principle already laid down, the occurrence of one hysteric fit, in a place of worship, will be exceeding likely to precipitate another, by abruptly breaking into the self-contemplative state. Moreover, if the preacher point out the person affected as one by whom saving grace has at that moment been received, as one predestined from eternity to conversion and final perseverance, the incident is eminently calculated to impress and convince his hearers, to add indefinitely to the power of his words, to arouse the most intense longing for a similar visitation, and to excite the most lively dread lest the grace sought for should be withheld. By both kinds of operation, hysteria is continually propagated and increased.

Upon recovering from the immediate effects of the paroxysm, the state of the "subjects" may admit of considerable variation. In some, even if not in many instances, the shock will be found to have dethroned the reason; and the unhappy patient will awake from somnambulism to fatuity or mania. In others, the original terror being completely removed by the assurances of the preacher, a state of feeling is induced, of which gratified vanity is the chief characteristic. The so-called penitent will converse fluently about her experience, describe her struggles and trials, her beatific visions, her eventual peace, her abiding assurance of salvation and eternal bliss, her profound repentance for her sins. She will seldom be ready to confess or bewail any particular transgressions; she will not be likely to afford any practical evidence of humility; and she will usually season her dish of marvels so as to suit the varying credulity of her different auditors. She is jealous of her position as the *prima donna* of her chapel or her sect, and elaborates ingenious novelties by which to crush the pretensions of any intrusive *débutante*. However ingenious, she will become wearisome at last. The nine days allotted to terrestrial wonders will pursue their inevitable course, and will bring in their train a girl who escaped, in her vision, from two devils instead of one; or who can garnish the narrative of her flight by incidents from the *Mysteries of London*, or the *Castle of Udolpho*. The first subject disappears from the scene, not to follow the example of Dorcas or of Lydia, but too often to lead a life concerning which her sometime hearers are glad to bury scandal beneath oblivion.

There remain other cases still, in which, in addition to mere terror, some real and actual sentiment of religion has been called forth. In these, somnambulism is induced but rarely; and the convulsive type of hysteria commonly prevails; so that the evil results immediately visible are not of so glaring a character as those already considered. They are, however, equally real; perhaps equally disastrous. Religion—capable as it is of uniting all the faculties of the mind in harmonious co-operation, but still pre-eminently a subject for the highest faculties, becomes in such persons inseparably associated and united with, and limited to, mere animal passion, that which, in the human race, is intended to stimulate the reason, not to supersede it. The ascendency freely given to all emotions connected with religion cannot be withheld from others; and, as already stated, a general surrender of the will to the passions is the result of such a violation of the order of nature. And, in many human creatures, the capacity for much passion lies dormant until discovered by events. The hysterical devotee of the class we are now considering is exemplary whilst untempted; but her house of faith rests only on the shifting quicksands of feeling. The first temptation that enlists any of the feelings on its side, that is associated with love, with hatred, with passion of whatever kind, at once attracts to itself all the forces by which it ought to be opposed. Of two opposite feelings, one must yield in action; and, where religion is only a feeling, it will be powerless against any other that may be more recently excited, or less easily displayed. On this principle, we conceive, may be explained may of the phenomena of reaction; and many of the more grievous lapses of persons of professing or reputed piety.

The "physical phenomena" of the revival having thus been shown to present, in their predisposing and exciting causes, in their progress, and in their results, a precise resemblance to the hysteria commonly seen in medical practice, and produced either by secular terrors or by amatory reverie, we are clearly entitled to consider them as belonging to the same family; and as being, in fact, very striking instances of morbid action. Without trespassing upon the domain of the theologian, we may regard these phenomena in their pathological relations; and may point out the methods by which they may be prevented, and the manner in which they may be overcome.

In the first place, however, we may observe that hysteria and Christianity are the zenith and nadir of the moral universe. God and Mammon are not more irreconcileable in their demands than a disease which has its very root and centre in indulged self-feeling, and a religion which prescribes love to others as the first and highest duty. Hysteria, indeed, is nothing but selfishness in its most concentrated and engrossing form; and, as such, is absolutely incompatible with the existence of Christian love in the heart, or with the relief which is afforded to all painful feelings by the sustaining and consoling influences of sincere religion.

It appears somewhat remarkable, therefore, at first sight, that hysteria should be so common a result of religious preaching among uneducated persons. During the last century alone (besides numerous examples in more ancient times), in Scotland, Wales, America, Ireland, and many parts of England, convulsive epidemics have been brought about by preaching—epidemics of which no precise or strictly medical history has been handed down; but which, from the scanty records preserved of them, appear to have been alike in all essential characters. It is scarcely less remarkable that no such disorders are recorded in connexion with the earthly ministry of our Lord; or as occurring during the apostolic era—notwithstanding that numerous very large assemblies then first had the Gospel preached to them. It is probable that some, at least, of the demoniacs who were then miraculously cured were what we should now call hysterical; but it may be regarded as certain that no convulsive seizures were the rule, or were frequent, or perhaps even ever occurred, among the early Christian converts. The fact would be far too important to be passed over in silence; and therefore the silence of the sacred writings may be held to be conclusive. There must be a reason for the difference in this respect between the teaching of apostolic and of modern times; and we are inclined to seek this reason in the nature of the tenets taught. The apostles preached a gospel of peace, of deliverance, of joy eternal and ineffable. Their successors in the ministry preach often a doctrine of damnation.

The truth made known to us by revelation (and almost ascertainable by reason), that the souls of the wicked, after death, go to their own place, and suffer there an inevitable misery, is a portion of the scheme of salvation necessary to be known, and highly proper to be taught; with the limitation that it should hold in the pulpit the same relative position that it holds in Scripture. A knowledge that eternity must be spent either in bliss or suffering is required, in order to vindicate to finite minds the justice of the Deity, and to afford a standard by which to estimate the pains and pleasures of the world. But it is incontestable that love to God, founded upon an assurance of God's love to man, is the only possible basis of Christian faith and duty; and it is inconceivable that love to God can ever be kindled at the flames of a literal hell. Denunciations of the wrath to come, frantic appeals to the terrors of a congregation, produce either hysteria or indifference—either shake the physical frame by positive dread of impending torture and mere selfish fears for personal safety, or else harden the listeners by the natural reaction of the human spirit against threats. In some of the more barbarous countries subject to the Greek Church, in Georgia and Mingrelia for instance, the priests' houses are commonly adorned by rude pictures, bearing a general resemblance to highly coloured illustrations of the *Ingoldsby Legends*, and representing the torments of the damned. In one popular cartoon, Georgian souls are depicted in a frying-pan, with a devil to turn them occasionally with a three-pronged fork, while another feeds the glowing fire beneath. In the background fresh souls are being received by other sable attendants; all having horns, hoofs, tails, and tridents *de rigueur*. Such pictures probably excite some consternation when they are new; but, long before they are old, the Georgians treat them as birds treat a scarecrow that they have found out. The Georgians cannot read, and their priests intend these pictures for sermons. They are precisely analogous to the efforts of the "loud-voiced man" whom Mr. Thackeray describes as "howling about hell-fire in bad grammar;" and, in either case, they very frequently express nothing but the longing of the artist or the preacher to persecute. Many a polemic would like to show the tender mercies of St. Dominic to all who differ from him; but is driven by want of power to vent his spite in words, and to dignify his words by the title of Christian doctrine.

We hold therefore, that these denunciations of the wrath to come, made prominent as the leading and essential feature of scriptural teaching—are without any shadow of justification or excuse. We do not here enter into their foundation in the word of God, but we maintain that the preachers of a certain school give them an undue prominence, and urge them at an unfitting season. We judge of them by their fruits. Hysteria, originating in terror, maintained for effect, terminating in profligacy or insanity, is a sad contrast to the peace that passeth understanding.

The pamphlet of the Archdeacon of Meath, to which attention was drawn in our last number, bears the testimony of an acute and impartial observer to the existence of a strong and deep current of religious feeling in the places to which the revival had then extended. Most earnestly do we trust that this current may deepen and widen as it flows; and that all who are brought within its influence may bear fruits meet for repentance. But in the hysterical phenomena, and, to a far greater extent, in the source from which they spring, we perceive an evil of frightful magnitude and incalculable strength. It is not only that a few women will fall into the gulf we have endeavoured to point out; or that many persons will be hardened by an almost instinctive repulsion of the teaching they will hear; but, above and beyond these evils, there is the setting up of a false standard of religion and morality—a standard under which self-consciousness will represent self-examination, ecstasy be accepted in lieu of faith, and convulsions as the fulfilment of Christian duty. We ascribe the origin of these and kindred delusions to the simple fact that the leaders of religious movements are usually ignorant of psychology, unacquainted with the more ordinary forms of hysteria, and therefore unable to discern the incompatibility of the conditions they excite with the feelings and changes to which they endeavour to refer them. They hope for the best in every instance; and they see no reason why spasms should be contrary to the spirit of sincere devotion.

The manner in which nervous attacks may be restrained during divine service, has been pointed out by Archdeacon Stopford; and his observations harmonize with the principle that, in order to control hysteria, its very existence should, as far as possible, be ignored. We conceive, however, that in times of religious excitement, and in the presence of a congregation of persons likely to be much swayed by feeling, the fear of exciting disease should never be absent from the mind of the preacher. In order to indicate the cautions which this fear should prompt, it is necessary to approach the subject of the proper place and office of emotion with regard to the reception of divine truth.

We have already stated that emotion, at once active and repressed, is the necessary precursor or essential cause of hysteria. It is plain that any conceivable emotion may be occasionally aroused under circumstances to render suppression of its manifestations desirable; but the rule is that those emotions only are repressed which would in general be thought blameworthy or indelicate. Practically, therefore, the causes of hysteria are reduced to the amatory passion, terror, anger or hatred, envy and vanity, either alone or variously blended; and the hysteria induced by preaching is traceable to terror and vanity almost exclusively. Fear of eternal suffering, and a wish to be distinguished as of the elect, are the ultimate elements of a "case" at a revival meeting.

Now the emotions appropriate to the Christian religion, excited by its great truths, and maintained by obedience to its precepts, are of a kind totally opposed to those mentioned above. Love to God and to our fellow-men, gratitude for blessings, and compassion for the afflicted and for sinners, are the states of feeling enjoined by the Divine Saviour, and these naturally expand into action and influence conduct, obtaining, as they do so, the approbation of all who experience their effects. Moreover, if under any (hardly conceivable) circumstances, they should be restrained for a time from their natural outward operation, they are not calculated to produce the agonizing sensorial tension that precedes hysteria, but rather to expand themselves upon the cerebrum, exciting intellectual instead of bodily activity, and engaging the mind about projects of usefulness or benevolence. In these feelings, the preacher who aims his teaching chiefly at the heart (as some, by reason of their natural tendencies and gifts, are prone to do), may find ample scope for all his efforts, especially when addressing those who are being urged to make their first faltering steps along the narrow way, and who require to be "allured" by the Divine hand, before, in the Valley of Achor, they find their "door of hope."

In dealing with such themes as these, it is most important, if not essential, that the preacher should not degrade them. The vocabulary in common use among certain classes of religionists has this tendency; and thus counteracts, in a marked degree, the elevating influence of the emotions proper to Christianity. The epithets that are sufficiently descriptive of a trinket or a baby, and the terms used about insipid or frivolous worldly enjoyments, when applied to the Divine Creator, or to the happiness of a blissful eternity, have a direct tendency to lower our conception of the Deity, and to make earthly things a standard for the things of heaven. To the extent that they do so, they affect the quality, so to speak, of the resulting emotions; and reduce them to the same level with kindred feelings excited by objects of a lower order. For example, in a case where love to a creature was the source of a temptation to violate a divine ordinance, and thus came into direct opposition with the impulse arising out of love to God, the relative power of the latter would be greatly increased by everything that tended to retain it habitually upon the highest level of the mind. Such an influence is exerted by reverence, by the constant employment, when speaking of the Deity, of words of the most exalted signification, and especially by a reverent avoidance of all unnecessary speech upon sacred topics. The contrary habits, the addiction to bald and disjointed chatter about religion, the application of familiar terms to the Creator, and the practice of deciding about the Divine providence as if it were not inscrutable, all illustrate, in the strong language of Bishop South, "the terrible imposture and force of words." They bring down God to a *quasi* humanity in the minds of those who practise them, and destroy the predominance, as motives, of the emotions which have God for their object.

With this precaution there is, perhaps, no limit to the degree in which the proper emotions of religion should be encouraged. They glide necessarily into appropriate action, filling the mind with pure and lofty thoughts, impelling the body to just and righteous deeds. They are the great sources of all abiding joy; and they furnish the only efficient consolation under every sorrow. They sustain the physical powers in a greater degree than any other kind of stimulant, and the energy they impart is succeeded by no depression. They have prompted the mightiest efforts of the intellect and the grandest decisions of the will, and they extend an impartial influence to all who will seek for their support; shielding the weak, guiding the strong, verifying the promises of hope, and withdrawing the sting of affliction.

The passion of terror, on the other hand, can only be safely used when it is rightly guided. It is often appealed to in education, and often in legislation, but always as the sanction of a rule that it is not difficult to keep. In such cases it is directed immediately into a practical channel of action or avoidance, and controls the conduct without injuring the health. In like manner, when terror is associated with the sanction of a divine command, the way to keep that command should be vigorously and strongly impressed upon the mind of the hearer; the more strongly, the less the individual is adapted, by nature or habits, for intellectual exertion. The most vivid imaginable fears about a future state, acting upon a philosophical mind, would discharge themselves on the cerebrum, would excite a spirit of inquiry, would enlist all the faculties of the organism in an endeavour to find the true answer to the question, "What shall I do to be saved?" It is quite conceivable that the course of thought and study thus suggested and initiated might receive the divine blessing, might in time become associated with higher motives than terror, with those motives which alone, as we are taught, can open the door of eternal bliss. It may often be right, therefore, in addressing an educated and sagacious audience, upon whom the glad tidings of the gospel appear inoperative, to open those sterner pages of Holy Writ which describe the doom of the condemned, and to direct attention to the fate of those who neglect to work out their own salvation. For the unthinking and the ignorant such an appeal to fear is not adapted; revelation, reason, and experience, all alike condemn it. Revelation, because the devils "believe and tremble;" and their actual state cannot be conducive to man's welfare. Reason, because the natural tendency of fear is to operate downwards upon the body, producing muscular movements, terminating in mere physical exhaustion. Experience, inasmuch as we have before us the records of many such appeals to communities or individuals, and the results of nearly all of them have been disastrous.

Upon this point, indeed, there is such an overwhelming mass of testimony, that we have been impelled to write strongly on the subject. At Belfast, there were great differences of opinion among the clergy with regard to the character of the cases "struck;" some being led by common sense, or observation, to consider that the influence thus manifested was pernicious; while others, animated, it may be presumed, by a less cautious zeal, and led astray, perhaps, by the hope that their own ministrations were especially blessed, laid themselves out to promote the occurrence of hysteria. On neither side does the question appear to have been viewed as one already decided by ample experience; but rather as one admitting of *à priori* argument, based upon hypotheses concerning the operation of the Holy Spirit. Our object has been to show that the bodily and psychical condition of "struck" cases is perfectly well understood, is reducible to known and simple laws of the animal economy, and may be summed up as that state of the human organism in which everything sensual and earthly is at the maximum of its power. There should no longer be any question or doubt whatever, either among the clergy or the public, as to the character of these seizures, or as to the duty and necessity of discountenancing and avoiding them; for although some of the cases are, we are told, now leading pure and holy lives, it is impossible for any one acquainted with the ordeal through which they have passed to think of its perils without a shudder, or to regard an escape from them otherwise than as a most especial mercy. Moreover, we cannot but view with apprehension the latitude that has been given to some of the chief causes of hysteria, and the probable prevalence of some of its milder, or less developed forms, in which painful emotions keep the consciousness turned inwards upon self, and lead to an instrospectiveness which is often mistaken for self-examination. Persons who are familiar with the æsthetic religionists of the high Tractarian school will have no difficulty in recognising the condition to which we refer.

As far as regards the present revival, we are glad to believe that the hysterical element once connected with it has almost or entirely perished. We do not believe that it ever gained any deep root, or that it was anything more than a result of an error of judgment on the part of the individual preachers who excited it. It was peculiar to no creed or sect, and was only so far localized that it followed an injudicious use of appeals to the terrors of an unenlightened audience. From the same source, in former times, similar disorders have sprung; either to overpower, or to be overpowered by, the religious character of the contemporaneous movements. We hold the two to be incompatible; and therefore, in the decline of hysteria, we recognise the best evidence that the work now proceeding is founded upon God's truth, and carried on in accordance with God's law. The testimony of moral improvement which we receive is in the highest degree encouraging; but, while the cause is so recent, the sceptical may question the durability of the effects; and, even with regard to the facts, there are rival statisticians in the field. In order, therefore, that the "revival" may maintain its ground in the minds of sober and Christian people, and in order that it may continue to receive, as it is now manifestly receiving, the divine blessing, too much caution cannot be exercised in the rejection of abnormal phenomena, too high a sense of responsibility cannot be entertained in examining successive phenomena as they are manifested. Our own faith forbids us to believe in any especial or unusual outpouring of the Holy Spirit; because we think that outpouring is normally continuous and all-sufficient. We conceive the revival to be man's work, man's act of turning to God, the result of man's yearning after spiritual communion with his Maker. From this point of view, we conceive that it may be blemished by human infirmities, or even altogether perverted by bad men or by demoniac agency; and on the other hand, that such agencies may be excluded by the exercise of an amount of care commensurate with the magnitude of the interests at stake. The more earnest the sharers in the movement, the more incessant and the more insidious will be the assaults upon their faith and practice; and the more jealous should be their watchfulness over the great charge committed to their keeping. Precisely in the degree in which such care and such watchfulness are exercised may we hope for the development of all good and the repression of all evil. Experience teaches us the possibility of an opposite result; and that a general turning of the people towards God is, in itself, the first step only; which may lead, if not duly followed up, to reactionary coldness and impiety. By this generation such a first step is now being made; and it is for each of us to strive, that our future movements may be directed towards our rightful goal. "Prove all things, hold fast that which is good." Such is the divine injunction; and a strict obedience to it will best promote what God vouchsafes to acknowledge as His glory, or to reveal to us as the means of man's salvation.

